# Iron as the Key Modulator of Hepcidin Expression in Erythroid Antibody-Mediated Hypoplasia

**DOI:** 10.1155/2014/421304

**Published:** 2014-12-18

**Authors:** J. C. Fernandes, P. Garrido, S. Ribeiro, P. Rocha-Pereira, E. Bronze-da-Rocha, L. Belo, E. Costa, F. Reis, A. Santos-Silva

**Affiliations:** ^1^Laboratory of Pharmacology and Experimental Therapeutics, IBILI, Faculty of Medicine, University of Coimbra, . 3000-548 Coimbra, Portugal; ^2^Institute for Molecular and Cellular Biology, University of Porto, 4150-180 Porto, Portugal; ^3^Department of Biological Sciences, Faculty of Pharmacy, University of Porto, Rua de Jorge Viterbo Ferreira No. 228, 4050-313 Porto, Portugal; ^4^Research Centre in Health Sciences, University of Beira Interior, 6201-001 Covilhã, Portugal

## Abstract

Erythroid hypoplasia (EH) is a rare complication associated with recombinant human erythropoietin (rHuEPO) therapies, due to development of anti-rHuEPO antibodies; however, the underlying mechanisms remain poorly clarified. Our aim was to manage a rat model of antibody-mediated EH induced by rHuEPO and study the impact on iron metabolism and erythropoiesis. Wistar rats treated during 9 weeks with a high rHuEPO dose (200 IU) developed EH, as shown by anemia, reduced erythroblasts, reticulocytopenia, and plasmatic anti-rHuEPO antibodies. Serum iron was increased and associated with mRNA overexpression of hepatic hepcidin and other iron regulatory mediators and downregulation of matriptase-2; overexpression of divalent metal transporter 1 and ferroportin was observed in duodenum and liver. Decreased *EPO* expression was observed in kidney and liver, while EPO receptor was overexpressed in liver. Endogenous EPO levels were normal, suggesting that anti-rHuEPO antibodies blunted EPO function. Our results suggest that anti-rHuEPO antibodies inhibit erythropoiesis causing anemia. This leads to a serum iron increase, which seems to stimulate hepcidin expression despite no evidence of inflammation, thus suggesting iron as the key modulator of hepcidin synthesis. These findings might contribute to improving new therapeutic strategies against rHuEPO resistance and/or development of antibody-mediated EH in patients under rHuEPO therapy.

## 1. Introduction

Erythropoietin (EPO), a 30.4 kDa glycoprotein, is a key hormone in the regulation of erythropoiesis, supporting proliferation, survival, and terminal differentiation of erythroid progenitor cells in the bone marrow [1, 2]. These effects are mediated through the interaction of EPO with its specific transmembrane receptor—erythropoietin receptor (EPOR) [3]. Acquired EPO deficiency has been associated with chronic kidney disease (CKD) and with chronic inflammatory diseases. Treatment with recombinant human erythropoietin (rHuEPO) achieves correction of these types of anemia; however, 5–10% of CKD patients develop resistance to the erythropoietic stimuli of rHuEPO. Several factors have been proposed to play a role in the development of this resistance, but the etiology of the impaired erythropoiesis, leading to worsening of the anemia or even to pure red cell aplasia (PRCA), is still unknown [4]. Casadevall et al. (1996) reported the presence of anti-EPO antibodies in a patient with transient PRCA, which functionally blocked the interaction between EPO and EPOR, thus resulting in impaired erythropoiesis [5]. Hara et al. (2008) also reported that serum from a patient with PRCA inhibited EPO-dependent cell proliferation, suggesting that the anemia was mediated by anti-EPO antibodies [6]. In several other cases, antibodies have been described in PRCA patients' sera that were selectively cytotoxic for marrow erythroid cells or were directed against EPO [7–9].

The current therapeutic use of rHuEPO to correct anemia in CKD patients has been associated with some cases of PRCA, due to the development of cross-reactive anti-EPO antibodies [10, 11]. Although this complication is very rare, the mechanisms underlying the break in immune tolerance to rHuEPO remain poorly clarified. The development of PRCA should be suspected in patients who have been under rHuEPO therapy for more than 3 months and develop a sudden severe unexplained fall in hemoglobin levels and reticulocytopenia and a marked decrease in bone marrow erythroblasts, despite continuing with rHuEPO treatment [12]. The diagnosis is confirmed by low serum EPO levels and detection of anti-EPO antibodies [13]. The production of anti-EPO antibodies and the inhibition of EPO-dependent erythroid cells are important mechanisms that might lead to erythroid hypoplasia (EH)/PRCA. The reduction in functional EPO affects the proliferative state of bone marrow erythroid cells, which will trigger several changes in iron metabolism, such as in serum iron, transferrin, and ferritin levels, among others [14–16].

Considering the scarce data in literature about how, facing a PRCA condition mediated by anti-EPO antibodies, the depression of marrow erythroid activity affects iron metabolism, our goal was to assess in male Wistar rats the effects of this condition on iron metabolism (iron absorption and iron traffic and storage) as well as on erythropoiesis. For that purpose, we performed hematological and biochemical studies to evaluate the erythropoietic and iron status and measured the expression of several mRNA-encoding erythropoiesis and iron metabolism regulating proteins on duodenum, liver, and kidney tissues.

## 2. Material and Methods

### 2.1. Animals and Experimental Protocol

**Figure 1:**
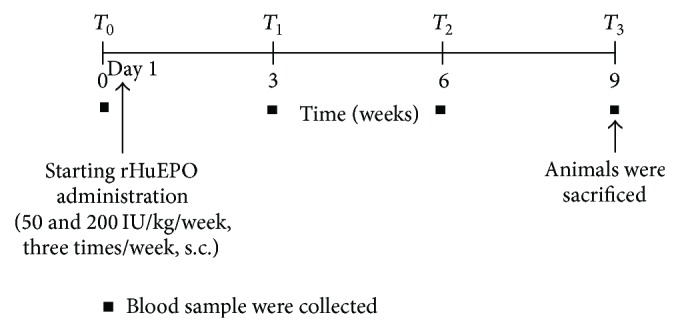
Experimental protocol.

Male Wistar rats (Charles River Lab. Inc., Barcelona, Spain), weighing 200–250 g, were maintained in an air-conditioned room, subjected to 12 h dark/light cycles, and given standard laboratory rat diet (IPM-R20, Letica, Barcelona, Spain) without iron supplementation and free access to tap water. Animal experiments were conducted according to the European Communities Council Directives on Animal Care. These procedures were in accordance with the Association for Pharmacology and Experimental Therapeutics and were approved by the Institutional Ethics Committee of the Faculty of Medicine from the University of Coimbra, approval ID: FMUC/04/13. The rats were divided into 3 groups (8 rats each): control: treated with saline solution; 50 IU rHuEPO: a therapeutic dose of 50 IU/Kg body weight (bw)/week of epoetin beta (Recormon-Roche Pharmaceuticals) treatment; 200 IU rHuEPO: treated with 200 IU/Kg bw/week of rHuEPO, a high dose, usually used on rHuEPO resistant patients. All the animals have completed the 9-week protocol (Figure 1).

### 2.2. Sample Collection and Preparation

#### 2.2.1. Blood

At the beginning of the experiments (*T*_0_) and at 3 (*T*_1_), 6 (*T*_2_), and 9 (*T*_3_) weeks after the first rHuEpo dose, the rats were subjected to intraperitoneal anesthesia with a 2 mg/kg bw of a 2 : 1 (v : v) 50 mg/mL ketamine (Ketalar, Parke-Davis, Lab. Pfizer Lda, Seixal, Portugal) solution in 2.5% chlorpromazine (Largactil, Rhône-Poulenc Rorer, Lab. Vitória, Amadora, Portugal). Blood samples were immediately collected by venipuncture, from the jugular vein, into vacutainer tubes without anticoagulant (to obtain serum) and with EDTA (to obtain whole blood and plasma) for hematological and biochemical studies; 3 mL blood samples were collected at *T*_0_, *T*_1_, and *T*_2_ to minimize interference with erythropoiesis mechanism; at the end of protocol (*T*_3_), a 10 mL sample was collected in order to perform all the biochemical and hematological assays.

#### 2.2.2. Tissues

At the end of the protocol, blood was collected and, afterwards, the rats were sacrificed by cervical dislocation; duodenum, liver, and kidneys were immediately removed, placed in ice-cold Krebs-Henseleit buffer, and carefully cleaned. A bone marrow aspirate from the femur was also performed.

### 2.3. Biochemical and Hematological Assays

Serum creatinine and urea concentrations were used as renal function indexes through automatic validated methods and equipment (Hitachi 717 analyser). Red blood cell (RBC), hematocrit, and hemoglobin (Hb) concentration were assessed in whole blood EDTA by using an automatic Coulter Counter (Beckman Coulter Inc., CA, USA); reticulocyte (RET) count was performed by microscopic counting on blood smears after vital staining with New Methylene Blue (Reticulocyte Stain, Sigma-Aldrich, St. Louis, MO, USA). Serum levels of erythropoietin were evaluated by rat specific ELISA kit (MyBioSource, USA). Serum iron concentration was determined using a colorimetric method (Iron, Randox Laboratories Ltd., UK), whereas serum ferritin and transferrin were measured by immunoturbidimetry (Laboratories Ltd., UK). Quantification of total bilirubin was performed by a colorimetric test (diazotized sulfanilic acid reaction, Roche Diagnostics); circulating levels of glucose and uric acid were determined by routine automated technology (ABX Diagnostics). Serum levels of interleukin-6 (IL-6), interferon-*γ* (IFN-*γ*), transforming growth factor (TGF-*β*1), and vascular endothelial growth factor (VEGF) were measured by rat-specific Quantikine ELISA kits from R&D Systems (Minneapolis, USA). High-sensitive C-reactive protein (hsCRP) was determined by using a rat-specific ELISA kit from Alpha Diagnostics International (San Antonio, USA).

### 2.4. Detection of Anti-EPO Antibodies

The detection of anti-EPO antibodies was carried out by ELISA, according to Urra et al., 1997, using rHuEPO (Recormon, Roche Pharmaceuticals) as antigen and, as secondary antibody, goat anti-rat IgG conjugated with horseradish peroxidase (Sigma; 100 ng/mL for 1 h, at room temperature) [17]. The substrate tetramethylbenzidine (TMB) (Sigma) was added and the reaction was stopped by the addition of sulphuric acid 1.25 mol/L. The optical density at 450 nm (OD450) was determined with an automatic plate reader.

### 2.5. Gene Expression Analysis

In order to isolate total RNA, 0.2 g of liver, duodenum, and kidney samples, from each rat, was immersed in RNA later (Ambion, Austin, USA) upon collection and stored at 4°C, for 24 h; afterwards, samples were frozen at −80°C. Subsequently, tissue samples weighing 50 ± 10 mg were homogenized in a total volume of 1 mL TRI Reagent using a homogenizer, and total RNA was isolated according to manufacturer instructions (Sigma, Sintra, Portugal). To ensure inactivation of contaminating RNAses, metal objects and glassware were cleaned with detergent, immersed in RNAse-free water (0.2% diethyl pyrocarbonate) for 2 h, and finally heated at 120°C for 1 h. RNA integrity (RIN, RNA Integrity Number) was analyzed using 6000 Nano kit, in Agilent 2100 bioanalyzer (Agilent Technologies, Walbronn, Germany), and 2100 expert software, following manufacturer instructions. The yield from isolation was from 0.5 to 1.5 *μ*g; RIN values were 7.8–9.0 and purity (A260/A280) was 1.8–2.0. The concentrations of the RNA preparations were confirmed with NanoDrop1000 (ThermoScientific, Wilmington, DE, USA). Possible contaminating remnants of genomic DNA were eliminated by treating these preparations with deoxyribonuclease I (amplification grade) prior to RT-qPCR amplification. Reverse transcription and relative quantification of gene expression were performed as previously described [18]. Real-time qPCR reactions were performed for the following genes:* EPO*,* EPOR*, transferrin receptor 2 (*TfR2*), hepcidin (*Hamp*), ferroportin (*SLC40A1*), Hemojuvelin (*HJV*), transferrin (*TF*), hemochromatosis (*Hfe*), divalent metal transporter 1 (*DMT1*), transferrin receptor 1 (*TfR1*), matriptase-2 (*TMPRSS6*), and bone morphogenic protein 6 (*BMP6*), which were normalized in relation to the expression of beta-actin (*Actb*) and 18S ribosomal subunit (*18S*), as well as to the mean of the control group. Primer sequences are listed in Table 1. Results were analyzed with SDS 2.1 software (Applied Biosystems, Foster City, CA, USA) and relative quantification was calculated using the 2^−ΔΔCt^ method [19]. In liver tissue, we studied the* EPO*,* EPOR*,* TfR2*,* Hamp*,* SLC40A1*,* HJV*,* TF*,* Hfe, BMP6,* and* TMPRSS6* gene expression; in duodenum tissue the gene expressions of* DMT1* and* SLC40A1* were studied, and in the kidney we evaluated the expression of* EPO* gene.

### 2.6. Data Analysis

For statistical analysis, we used the Statistical Package for Social Sciences (SPSS), version 22.0. Results are presented as mean ± standard deviation. Multiple comparisons between groups were performed by one-way ANOVA supplemented with Tukey's HSD post hoc test. For single comparisons, we used the Mann-Whitney *U* test. Significance was accepted for a *P* minor than 0.05.

## 3. Results

**Figure 2:**
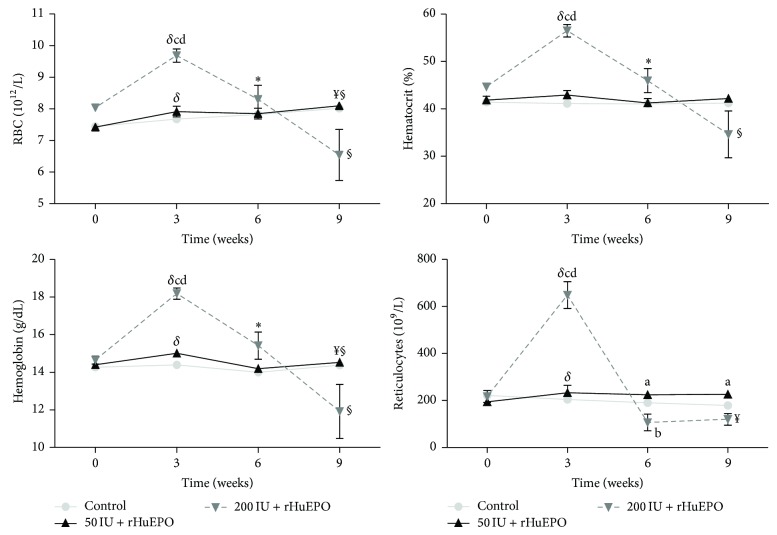
Erythrocyte data and reticulocyte count during the follow-up period of 9 weeks under rHuEPO treatment. Results are expressed as mean ± SD. ^a^ *P* < 0.05 versus control; ^b^ *P* < 0.05 versus 50 IU rHuEPO; ^c^ *P* < 0.001 versus control; ^d^ *P* < 0.001 versus 50 IU rHuEPO; ^*δ*^ *P* < 0.005  *T*_0_ versus *T*_1_; ^*^ *P* < 0.05  *T*_1_ versus *T*_2_; ^§^ *P* < 0.05  *T*_2_ versus *T*_3_.

The changes in RBC count, Hb concentration, hematocrit, and the number of reticulocytes for the three groups are presented in Figure 2. During the 9-week experimental protocol, the 50 IU rHuEPO group, when compared to the control group, showed similar values for RBC count, Hb concentration, hematocrit, and reticulocytes, though a significant increase in reticulocyte count was observed at *T*_2_ and *T*_3_. Concerning the rats treated with 200 IU rHuEPO, in the first 3 weeks, we found a significant increase (>30% the basal value) in hemoglobin levels, RBC count, hematocrit, and reticulocyte count, as compared to control and 50 IU rHuEPO groups; after this period, the hemoglobin concentration, hematocrit, and RBC count as well as the number of reticulocytes decreased, reaching significantly lower values at *T*_2_, as compared to *T*_1_; this trend towards decreasing values was maintained till the end of the experiment (9 weeks). At the end of the protocol, serum erythropoietin levels were similar among the three groups (Table 2). No changes were observed in glucose, creatinine, urea, uric acid, and bilirubin levels for both rHuEPO groups along the entire protocol, when compared to the control. The inflammatory marker, IL-6, presented a significant decrease in the 200 IU rHuEPO group when compared with the control and 50 IU rHuEPO groups; hsCRP, IFN-*γ*, TGF-*β*1, and VEGF showed no significant differences between the three studied groups. Concerning iron metabolism, we found that serum iron was significantly higher for the 200 IU rHuEPO group, when compared with the control and 50 IU rHuEPO groups; no significant differences were found in ferritin and transferrin serum levels between the three studied groups (Table 2).

Serum samples from all animals were also analyzed for anti-rHuEPO antibodies at *T*_1_, *T*_2_, and *T*_3_; however, only at *T*_3_ we detected the presence of anti-rHuEPO antibodies in circulation. These were detected in 5 rats under 50 IU rHuEPO treatment, as well as in 5 rats treated with 200 IU rHuEPO (62.5% for both groups); the antibody titer was fourfold higher in the group under 200 IU rHuEPO; the latter presented a titer of 1 : 16, and the group under the lower rHuEPO dose presented a titer of 1 : 4. Within each rHuEPO group, no other significant differences were found between the rats with or without anti-rHuEPO antibodies.

Bone marrow examination of rats treated with 200 IU rHuEPO showed a proportion of red-cell precursors significantly lower than those without rHuEPO treatment. The myeloid : erythroid ratio was 2.1 : 1 for the control group, while for the 200 IU rHuEPO group the ratio was 6.8 : 1; the 50 IU rHuEPO group presented a myeloid : erythroid ratio similar to that found for the control (2.5 : 1).

**Figure 3:**
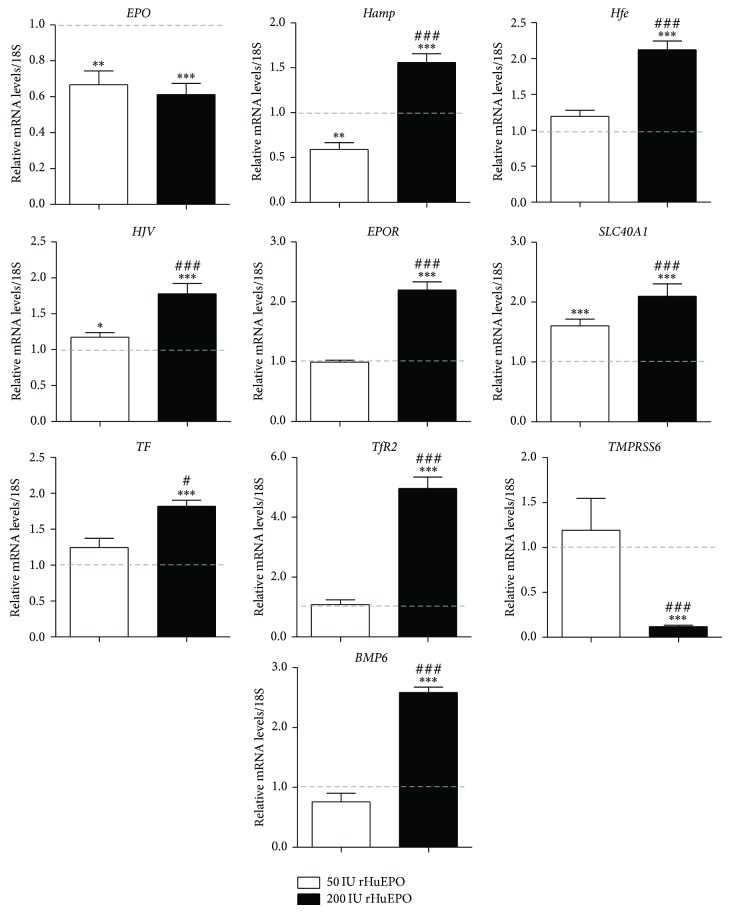
Relative mRNA expression of erythropoietin and iron regulatory proteins in the liver, at the end of the protocol (9 weeks). 18S rRNA was used as reference gene. Results are expressed as mean ± SD. ^*^ *P* < 0.05, ^**^ *P* < 0.01, and ^***^ *P* < 0.001 versus control group; ^#^ *P* < 0.05, ^##^ *P* < 0.01, and ^###^ *P* < 0.001 versus 50 IU rHuEPO.

Major changes were observed in the gene expression of iron regulatory proteins and in erythropoietic regulatory proteins in liver tissue (Figure 3), particularly in the 200 IU rHuEPO group. Indeed, the 50 IU rHuEPO group showed significant overexpression of* HJV* and* SLC40A1 *genes, as compared to control, while the 200 IU rHuEPO group presented significant overexpression of* Hamp, Hfe, HJV, EPOR*,* SLC40A1, Tf, TfR2, *and* BMP6* genes, as compared to control and 50 IU rHuEPO group.* EPO *gene was significantly downregulated in both groups, and* TMPRSS6 *gene expression was significantly downregulated only in the 200 IU rHuEPO group, as compared to the control and 50 IU rHuEPO groups.

**Figure 4:**
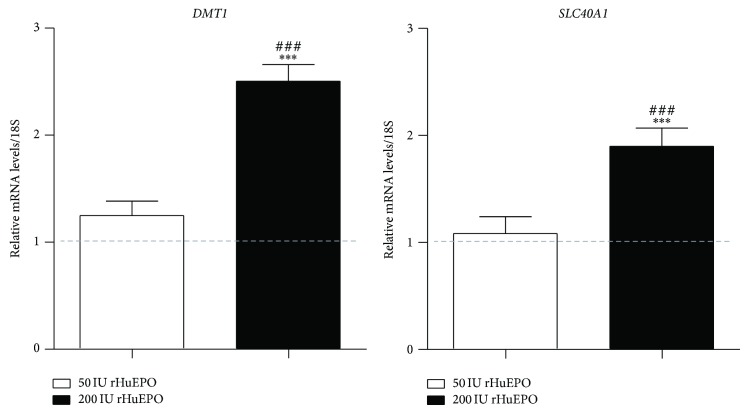
Relative mRNA expression of SLC40A1 and DMT1 in the duodenum, at the end of the protocol (9 weeks). 18S rRNA was used as reference gene. Results are expressed as mean ± SD. ^***^ *P* < 0.001 versus control group, ^##^ *P* < 0.01, and ^###^ *P* < 0.001 versus 50 IU rHuEPO.

**Figure 5:**
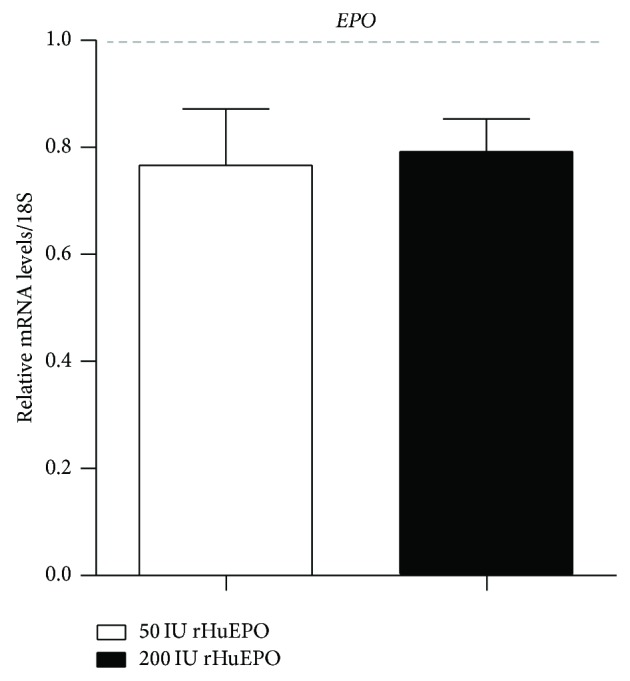
Relative mRNA expression of erythropoietin in the kidney, at the end of the protocol (9 weeks). 18S rRNA was used as reference gene. Results are expressed as mean ± SD.

The evaluation of the expression of* DMT1* and* SLC40A1 *genes in duodenum showed that only the 200 IU rHuEPO group presented a significant upregulation for both genes (Figure 4), as compared to the control. Furthermore, as observed in liver, the expression of* EPO* in kidney tissue presented a trend towards downregulation in both rHuEPO groups, when compared to the control (Figure 5).

## 4. Discussion

Antibody-mediated EH/PRCA is a rare pathological complication associated with the use of recombinant human erythropoietins and development of anti-EPO antibodies. However, the use of EPO biosimilars has increased the number of cases in the last years [11, 20, 21]. Clinically, that condition not only abrogates the effect of the rHuEPO, but may also neutralize endogenous EPO, leading to severe EH and transfusion-dependent anemia.

In this work we developed an animal model of antibody-mediated EH, by long-term treatment with a high dose of rHuEPO (200 IU/Kg bw/week during 9 weeks) in Wistar rats. In order to confirm that we were in fact in the presence of an animal model of this hematological disorder, we assessed the parameters typically used as diagnostic criteria for EH/PRCA, which include normocytic anemia with sudden onset, normocellular bone marrow with a selective reduction in red blood cell precursors beyond proerythroblasts, reticulocytopenia, normal or slightly decreased leucocyte counts, and increased serum iron [22]. We found that the group treated with 200 IU/Kg bw/week of rHuEPO along 9 weeks fits all these criteria and the majority (65%) of the rats presented detectable anti-rHuEPO antibodies, suggesting that we achieved an antibody-mediated EH. The group under 50 IU/Kg bw/week rHuEPO treatment presented only slight changes, as compared to control; however most of the rats (65%) presented anti-rHuEPO antibodies at a lower titer (four times less), suggesting that a longer experimental protocol could also lead to the changes associated with EH.

Indeed, Casadevall (2005) reported that the detection of anti-erythropoietin antibodies in circulation is not immediately associated with PRCA [23]. Moreover, no significant changes were observed in hsCRP, IL-6, and IFN-*γ*, for both rHuEPO treated groups, showing that no inflammatory changes could interfere with erythropoiesis and iron metabolism.

Our data suggest that the development of EH was due to the antibodies directed to rHuEPO that inhibited the erythropoietic stimuli of both rHuEPO and endogenous EPO. Indeed, in spite of the normal endogenous EPO levels that were similar to those of the control group, the 200 IU rHuEPO group developed anemia, suggesting that the anti-rHuEPO antibodies also neutralized the action of endogenous EPO. It has been described that the presence of rHuEPO in circulation may increase severalfold the expression of* EPOR* [4]. Actually, we found an overexpression of* EPOR* in the liver of the 200 IU rHuEPO group. The decreased* EPO* expression observed in kidney and liver tissues, for both rHuEPO groups, seems to be in accordance with a physiological response to the increased circulating levels of rHuEPO, used in the treatment. However, we should not exclude the hypothesis raised by Piron et al. (2001), that this anemia could be due to an inappropriate erythropoietic stimulation or to intrinsic failure of erythroid cells to respond to that stimulus [24].

Anemia usually triggers erythropoiesis by increasing EPO production through the hypoxia-inducible factor (HIF) pathway, by mobilizing iron from the iron storage pool and by increasing iron absorption, in order to face the increased iron needs for erythropoiesis [25]. Considering that, in antibody-mediated EH, the erythropoietic stimuli fail, due to the inhibition of rHuEPO by anti-rHuEPO antibodies, the serum iron should increase, as its absorption and mobilization would be triggered by the anemic condition, and hepcidin should be repressed to favour iron absorption and mobilization. Actually, we found a significant increase in serum iron in the 200 IU rHuEPO group. It is known that increasing serum iron induces hepcidin synthesis by a complex pathway. Indeed, hepcidin, encoded by the* Hamp* gene, is believed to play a key regulatory role in iron absorption. It controls plasma iron concentration and tissue distribution of iron by inhibiting intestinal iron absorption, iron recycling by macrophages, and iron mobilization from stores [26]. Hepcidin acts by inhibiting cellular iron efflux in hepatocytes, enterocytes, and macrophages, through binding to ferroportin, inducing its degradation [27]. Hepatic* Hamp* expression is regulated by a cohort of proteins, including Hfe, TfR2, HJV, BMP6, matriptase-2, and transferrin [28–30]. Studies in both human and animal models indicate that* Hfe* and* TfR2* are mild inducers of hepcidin expression [28], as compared to* HJV* and* Tmprss6*, which are robust modifiers of hepcidin expression [31–33]. TfR2 has been hypothesized to act as a sensor for iron levels in the body because of its largely hepatocyte-specific expression and its ability to mediate cellular iron transport [34]. Although TfR2 is a minor contributor to the uptake of transferrin-bound iron by the liver, experiments with mice have shown that its major role is to modulate the signalling pathway that controls* Hamp* induction [34].* Hfe* has also been implicated in the iron signalling complex that modulates hepcidin transcription by sensing changes in iron levels [35]. Increasing expression of* HJV* enhances* Hamp* expression by interacting with BMPs [36] possibly as a coreceptor. Matriptase-2 has been shown to negatively regulate* Hamp* gene and, therefore, to decrease hepcidin expression and promote iron uptake [37]. It is highly expressed in the liver and participates in a transmembrane signaling pathway triggered by iron deficiency and suppresses hepcidin expression by cleaving membrane-bound hemojuvelin (mHJV) to increase iron absorption [38].

**Figure 6:**
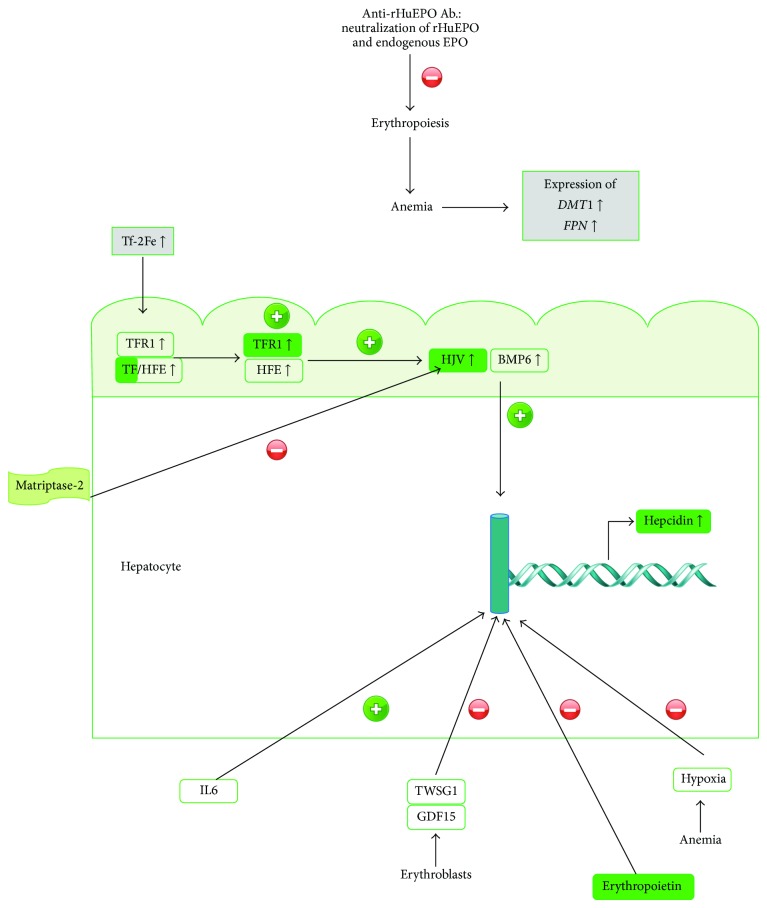
Model proposed for erythropoiesis and iron metabolism in erythroid antibody-mediated hypoplasia. Anti-rHuEPO antibodies inhibit both rHuEPO and endogenous EPO leading to anemia that is further aggravated by increased iron that favours hepcidin expression.

In the liver, diferric transferrin competes with TfR for binding to Hfe, and, when iron is increased, more Hfe is available to bind to TfR2; this complex, TfR2-Hfe, promotes HJV binding to BMP6, increasing hepcidin synthesis [34, 36]. Indeed, we found that increased serum iron in the 200 IU rHuEPO group was associated with an overexpression of* Tf, TfR2, BMP6, Hfe, *and* HJV* in the liver and, accordingly, with an overexpression of* Hamp*. Moreover, a downregulation in matriptase mRNA was observed in the liver that might further contribute to the overexpression of hepcidin (Figure 6). In accordance with the overexpression of hepcidin, a reduction in serum iron would be expected, due to the degradation of ferroportin by hepcidin, instead of the increase that we found. It is known that regulation of iron absorption is mediated by signals reflecting oxygen tension in enterocytes, intracellular iron levels, and systemic iron needs. Enterocyte oxygen tension regulates iron absorption through its effects on the transcription of* HIF*s and subsequent changes in transcription of* DMT1* [39]. Iron exits the enterocyte through the efflux transporter ferroportin 1 (FPN), the only member of the SLC40 family of transporters and the first reported protein that mediates the exit of iron from cells [40]. Actually, we found an overexpression of DMT1 and ferroportin in the duodenum and liver from the 200 IU rHuEPO group, in response to the anemic state. However, as the overexpression of hepcidin compromises iron absorption and mobilization, the observed increase in serum iron might result mainly from the decreased use of iron for the inhibited erythropoiesis.

Hypoxia, EPO, as well as twisted gastrulation protein 1 (TWSG1) and growth differentiation factor 15 (GDF15) [41], both produced by erythroblasts, is known to downregulate hepcidin synthesis. Considering the inhibition of erythropoiesis through the anti-rHuEPO antibodies, the production of TWSG1 and GDF15 might be reduced and, therefore, migh be unable to downregulate hepcidin synthesis. We also found that rHuEPO treatment did not alter renal function and glucose levels and was not associated with an inflammatory process, when compared to the control.

Considering the four functionally hepcidin regulatory pathways described [42–45], erythropoiesis, iron status, oxygen tension, and inflammation, we found that, in our model, erythropoiesis was blunted leading to anemia, iron was increased, and there was no inflammation. It seems, therefore, that the increased iron concentration is the key modulator of hepcidin synthesis in this type of erythroid hypoplasia. In fact, it has been recently reported that the reduction of serum iron by iron chelation therapy promoted an improvement in erythropoiesis in PRCA, though the mechanism whereby this is achieved is still unclear [46].

In conclusion, our data suggest that, in the case of erythroid antibody-mediated hypoplasia/PRCA induced by a high rHuEPO dose (200 IU), as erythropoiesis is blunted through anti-rHuEPO antibodies, iron concentration becomes the key modulator for hepcidin synthesis, which will, probably, contribute to further aggravation of the anemia. These findings might be important to improve new therapeutic strategies against rHuEPO resistance and/or development of antibody-mediated EH/PRCA in patients under rHuEPO therapy.

## Figures and Tables

**Table 1:** List of primer sequences (F: forward; R: reverse).

Gene	Primer sequences
*EPO *	F: 5′-AGGGTCACGAAGCCATGAAG-3′
	R: 5′-GAT TTC GGC TGT TGC CAG TG-3′

*EPOR *	F: 5′-GCG ACT TGG ACC CTC TCA TC-3′
	R: 5′-AGT TAC CCT TGT GGG TGG TG-3′

*Hamp *	F: 5′-GAA GGC AAG ATG GCA CTA AGC-3′
	R: 5′-CAG AGC CGT AGT CTG TCT CG-3′

*TfR2 *	F: 5′-CAA GCT TCG CCC AGA AGG TA-3′
	R: 5′-CGT GTA AGG GTC CCC AGT TC-3′

*SLC40A1 *	F: 5′-CAG GCT TAG GGT CTA CTG CG-3′
	R: 5′-CCG AAA GAC CCC AAA GGA CA-3′

*HJV *	F: 5′-GCC TAC TTC CAA TCC TGC GT-3′
	R: 5′-GGT CAA GAA GAC TCG GGC AT-3′

*TF *	F: 5′-GGC ATC AGA CTC CAG CAT CA-3′
	R: 5′-GCA GGC CCA TAG GGA TGT T-3′

*Hfe *	F: 5′-CTG GAT CAG CCT CTC ACT GC-3′
	R: 5′-GTC ACC CAT GGT TCC TCC TG-3′

*DMT1 *	F: 5′-CAA CTC TAC CCT GGC TGT GG-3′
	R: 5′-GTC ATG GTG GAG CTC TGT CC-3′

*TfR1 *	F: 5′-GCT CGT GGA GAC TAC TTC CG-3′
	R: 5′-GCC CCA GAA GAT GTG TCG G-3′

*TMPRSS6 *	F: 5′-CCG AAT ATG AGG TGG ACC CG-3′
	R: 5′-GGT TCA CGT AGC TGT AGC GG-3′

*BMP6 *	F: 5′-GCT GCC AAC TAT TGT GAC GG-3′
	R: 5′-GGT TTG GGG ACG TAC TCG G-3′

*18S *	F: 5′-CCA CTA AAG GGC ATC CTG GG-3′
	R: 5′-CAT TGA GAG CAA TGC CAG CC-3′

*Actb *	F: 5′-GAG ATT ACT GCC CTG GCT CC-3′
	R: 5′-CGG ACT CAT CGT ACT CCT GC-3′

**Table 2:** Hematological and biochemical data at the end of protocol (9 weeks).

Parameters	Groups		
	Control	50 IU rHuEPO	200 IU rHuEPO
EPO (mIU/mL)	1.87 ± 0.25	1.65 ± 0.23	1.80 ± 0.21
Iron (*µ*g/dL)	183.63 ± 41.58	188.63 ± 35.27	255.14 ± 112.28^a,b^
Ferritin (ng/dL)	165.13 ± 19.19	168.50 ± 42.95	162.57 ± 39.81
Transferrin (mg/mL)	97.75 ± 8.68	99.38 ± 6.59	96.14 ± 9.68
Glucose (mg/dL)	160.88 ± 29.04	174.62 ± 20.21	187.71 ± 37.43
Creatinine (mg/dL)	0.40 ± 0.05	0.37 ± 0.03	0.35 ± 0.06
Urea (mg/dL)	45.10 ± 1.92	45.05 ± 3.56	42.74 ± 5.10
Uric acid (mg/dL)	0.94 ± 0.46	0.70 ± 0.13	1.03 ± 0.52
Bilirubin (ng/mL)	0.043 ± 0.016	0.054 ± 0.013	0.064 ± 0.014
IL-6 (pg/mL)	138.10 ± 5.34	139.98 ± 6.12	130.16 ± 5.06^a,b^
hsCRP (*µ*g/mL)	229.31 ± 24.55	237.49 ± 28.92	249.46 ± 33.49
IFN-*γ* (pg/mL)	24.44 ± 23.07	21.45 ± 6.80	20.82 ± 10.13
TGF-*β*1 (ng/mL)	80.21 ± 4.99	80.03 ± 17.22	78.10 ± 5.55
VEGF (pg/mL)	5.36 ± 4.26	5.84 ± 3.85	6.04 ± 3.25

Results are expressed as mean ± SD. ^a^ *P* < 0.05 versus control; ^b^ *P* < 0.05 versus 50 IU rHuEPO. IL-6: interleukin-6; hsCRP: high-sensitive C-reactive protein; IFN-*γ*: interferon-*γ*; TGF-*β*: transforming growth factor beta1; VEGF: vascular endothelial growth factor.
